# Partial Purification and Characterization of Anticoagulant Factor from the Snake (*Echis Carinatus)* Venom

**Published:** 2013-11

**Authors:** Elham Amrollahi Byoki, Abbas Zare Mirakabadi

**Affiliations:** 1 Payam Noor University of Tehran, Tehran, Iran; 2 Department of Venomous Animals and Antivenom Production, Razi Vaccine and Serum Research Institute, Karaj, Iran

**Keywords:** Anticoagulant factor, Chromatography, *Echis carinatus*, Snake venom

## Abstract

***Objective(s):*** Snake venoms contain complex mixture of proteins with biological activities. Some of these proteins affect blood coagulation and platelet function in different ways. Snake venom toxin may serve as a starting material for drug design to combat several pathophysiological problems such as cardiovascular disorders. In the present study, purification of anticoagulation factor from venom of snake (*Echis carinatus*) was studied.

***Materials and Methods:*** Anticoagulation activity of crude venom, fractions and purified peptide were determined by using prothrombin time (PT) and thrombin time (TT). Three fractions were partially purified from the venom of *E. Carinatus* by gel filtration on sephadex G-75 and final purification was performed by high-performance liquid chromatography (HPLC) with C18 column. A purified anticoagulant factor was derived which showed a single protein band in SDS-PAGE electrophoresis under reducing condition.

***Results:*** Results of PT and TT tests for purified peptide (EC217) were found to be 102±4.242 and < 5 min. respectively. Determination of molecular weight revealed that the active purified peptide (EC217) was about 30 KD.

***Conclusion:*** The present study showed that the venom of *E. carinatus *contains at least one anticoagulant factor.

## Introduction

Snake venoms are complex mixture of pharmacologically active proteins and polypeptides (1, 2). Over the years, a number of toxins that affect the blood circulation have been isolated and characterized from various snake venoms (3–6). These studies have helped researchers to develop different new therapeutic agents for the treatment of cardiovascular and hematological disorders (7, 8). Venom proteins affecting blood coagulation can functionally be classified as pro-coagulant or anticoagulant proteins on the basis of their ability to shorten or prolong the process of coagulation (9). Anticoagulant factors isolated from *Echis carinatus* were included of Echicetin, Echsitatin, etc (10-12). Echicetin was heterodimeric protein from the venom of the saw-scaled viper*, Echis carinatus *(13). It binds to platelet glycoprotein Ib (GPIb) and therefore inhibits platelet aggregation. Echistatin another active peptide which inhibits the platelet aggregation was purified from the venom of *E.carinatus *(14). An acidic phospholipase A2 has been purified from the Indian *E. carinatus* has a molecular weight of 16000 by SDS-PAGE (15). The Iranian *E. carinatus *is one of the most dangerous species among the viper snakes. Its venom cause severe blood coagulation manifestations in patients (16). In this study, we report the purification and characterization of an anticoagulation protein from the venom of this snake species, *E. carinatus *that exhibits anticoagulant activity on human blood.

## Materials and Methods

***Chemicals***

Sephadex G-75 and C18 columns were purchased from Pharmacia Biotech Company (Sweden). Bovine serum albumin, the kit of standard protein markers and other reagents for biochemical assays were purchased from Merk Company (Germany). All other reagents were analytical grade chemicals.

***Venom collection***

The *E. carinatus *snake (local name *Jaafari*) was identified and confirmed through morphometric index by experts in Razi Vaccine and Serum Research Institute, Iran. Venom was extracted by allowing the snake to bite into a para-film stretched over a disposable plastic cup. The Venom was frozen at –50°C and then lyophilized.

***Protein determination***

Protein concentration was measured by the method of Lowry *et al *(17), using bovine serum albumin (BSA) as standard.

***Crude venom fractionation***

Lyophilized crude venom of *E. carinatus *(250 mg) was dissolved in 3 ml of 0.1 M ammonium acetate buffer (pH 7.4) and centrifuged at 5,000 rpm for 15 min at 4°C and was filtered by 0.45 microfilter to remove the insoluble materials. The clear supernatant was applied on gel chromatographic column (150 x 2 cm) of Sephadex G-75, previously equilibrated with the ammonium acetate buffer (pH 7.4) and then eluted with the same buffer. Fractions of 10 ml/tube were collected at a flow rate of 60 ml/hr at 4°C. The obtained fractions were denominated as EC1 to EC3, indicating *E. carinatus *fractions 1 to 3. 

***Purification of anticoagulant factor***

For the purification of venom fractions, a reverse phase HPLC was used with C18 column. Elution was then carried out with a linear gradient of water (solution A) and acetonitrile (solution B) added 0.1% TFA. The flow rate was adjusted at 0.5 ml/min. Protein absorption was monitored at 280 nm. Peaks were collected manually and lyophilized. 

The relative abundances (% of the total venom proteins) of the different protein families in the venom were estimated from the relation of the sum of the areas of the reverse-phase chromatographic peaks containing proteins from the same family to the total area of venom protein peaks.

***Sodium dodecyl sulfate-polyacrylamide gel electrophoresis (SDS-PAGE)***

Purified venom fraction (20 μg) along with crude venom (200 μg) and standard proteins were electrophoresed on SDS-PAGE. It was performed by the method of Laemmli (20) and the gels were stained using silver stain and coomassie brilliant blue. For determination of molecular weight SDS-PAGE of 15% with a molecular weight standard ranging from 6.5 to 200 kDa was used. 

***Prothrombin time (PT) assay***

Normal pooled plasma was obtained from ten individual healthy donors, without history of bleeding or thrombosis. Blood was then centrifuged for 20 min at 2,400 rpm and the plasma was fresh when used. 

Prothrombin time reagent (200 μl) and sample aliquots (200 μl) containing 100, 10 and 5 µg as protein for crud venom fractions and purified peptide were pre-incubated for 10 min at 37°C and mixed for five seconds and then 100 μl of plasma was added and clotting time was recorded (18). One unit of anticoagulant activity corresponds to an increase of 20 sec in normal plasma coagulation called as International Normalized Ratio (INR) and calculated as follows.

$\mathrm{INR}={\left(\frac{{\mathrm{PT}}_{\mathrm{test}}}{{\mathrm{PT}}_{\mathrm{normal}}}\right)}^{\mathrm{ISI}}$

ISI or International Sensitivity Index was 1 for the kit used in the present study. The PT test was carried out for crud, fractions from gel filtration and purified active protein.

***Fibrinolytic assay***

Fibrinolytic activity of crude venom, fractions and purified peptide (EC217) was measured using a procedure modified from Bajwa *et al* (1980). Fibrinogen solution from human plasma (9.4 mg/ml, 300 AL, SIGMA) and thrombin from bovine plasma solution (38.5 U/ml, 10 AL, SIGMA) were added to each well of plate. The plate was shaken gently and the solution allowed clotting at room temperature. The plate was then incubated for 3 hr, at 37°C Twenty microliters of crude venom (1 mg/ml) as well as its fractions (1 mg/ml) were added individually in the center of their corresponding wells, and incubated overnight, at 37°C. Seven hundred microliters of 10% trichloroacetic acid (TCA) was placed in each well, and decanted after 10 min. If a zone of clearing was observed, the assay was considered positive. The assay was repeated three times for each sample (19).

## Results

***Protein determination*** ***of E. carinatus crude venom***

Protein concentration of crude venom was measured by the method of Lowry. The total protein was found to be 230 mg of 250 mg crude venom by dry weight which was about 92% protein components in general (Table 1).

***Prothrombin time (PT) assay on crude venom***

The anticoagulant activity of *E. carinatus *venom was determined using prothrombin time assay. The results of this study show that *E. carinatus* venom affected the prothrombin time and thrombin time by delayed plasma coagulation (Table 1). The crud venom at concentration of 100 µg has completely prevented the coagulation. These observations indicate that EC2 has the significant anticoagulant activity with a prolonged clotting time of 27.12±6.99 sec which was approximately 2 folds greater than control plasma with an average clotting time of 11±1.19 sec.

***Isolation of anticoagulant factor***

**Figure1:**
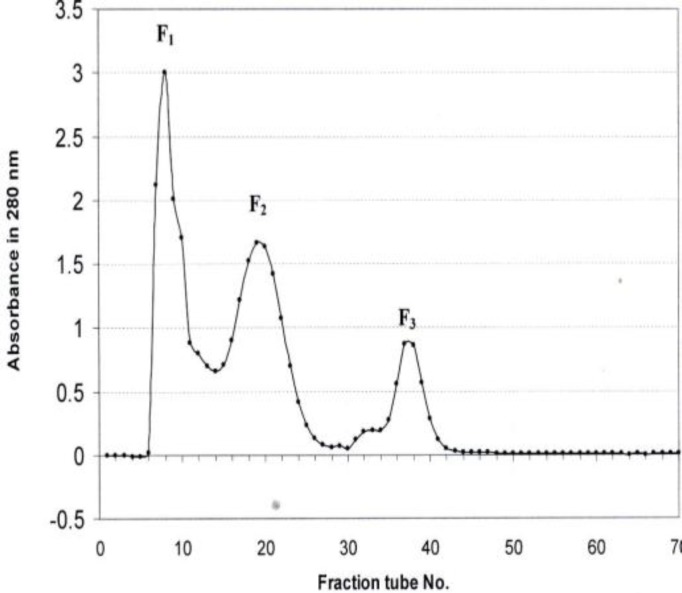
Sephadex G-75 chromatography of Iranian snake (*Echis carinatus*) venom

**Figure2:**
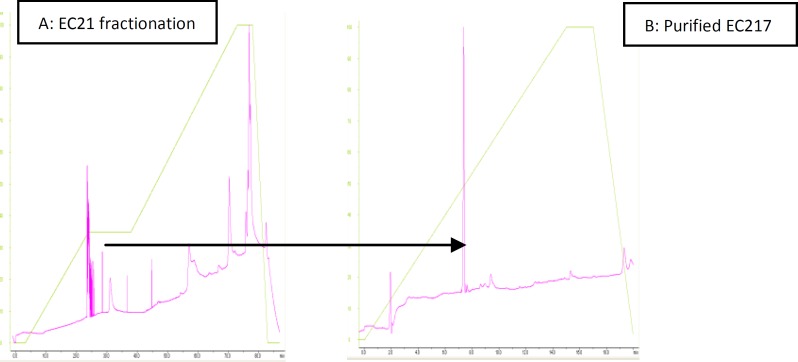
**A: **Further purification of fraction EC2 obtained from Gel filtration by HPLC using gradient of acetonitril and water. **B: **Purity assay of active peptide 217 as anticoagulant factor by HPLC using same gradient of acetonitril and water at absorbance of 280 nm

To purify the anticoagulant protein, in the initial Sephadex G-75 fractionation of the crude *E. carinatus *venom as shown in Figure 1, three peaks were obtained (EC1 to EC3). When all the fractions were tested for anticoagulation, second fraction (EC2) showed anticoagulant activity with increase in clotting time from 12 sec to 27 sec as determined by prothrombin time assays. Therefore, EC2 fraction was used for further purification of anticoagulant factor in this study. Further purification was carried out by HPLC using C18 column (Figure 2). The results revealed 54 fractions out of which EC217 suggested the anticoagulant activity with INR of 9.466±0.387 in PT test (Table2).

***Purity and determination of molecular weight***

**Figure 3:**
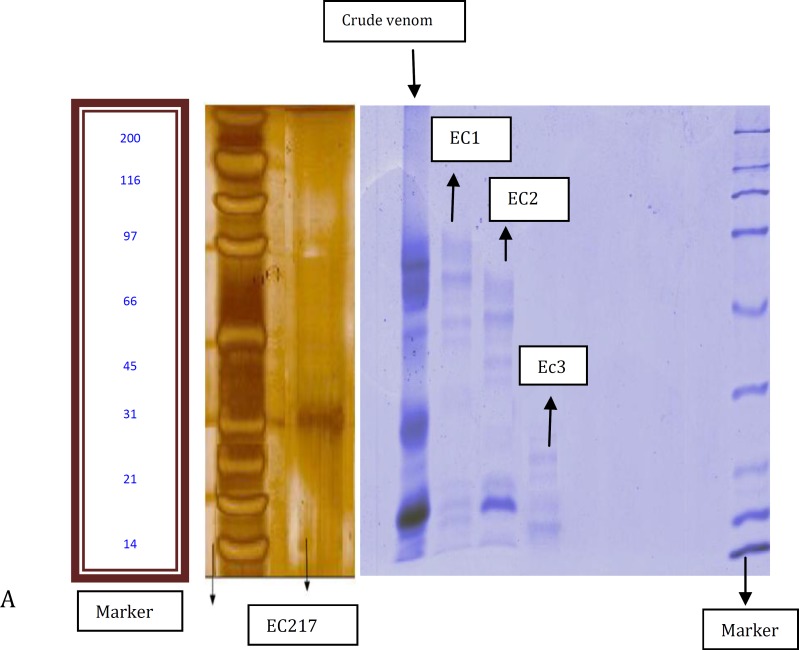
Electrophoresis of crude venom gel filtration fractions and EC217 active anticoagulant peptide of *Echis carinatus* venom. **A:** From left to right; marker and EC217 respectively. Stained with silver nitrate. **B: **From left to right; crude venom, EC1, EC2, EC3 fractions and markers respectively. Gel was stained with coomassie brilliant blue. Both of gels analyzed on 15% SDS-polyacrylamide gel in the presence of 1% 2- mercaptoethanol

Figure 2 A showing the protein bands in crude venom as well as various protein fractions isolated during gel filtration. Purity of protein EC217 was confirmed by SDS-PAGE which showed single band in SDS-PAGE stained by silver nitrate. Isolated anticoagulant factor (EC217) also indicated the high purity as analyzed by C18 reverse phase HPLC (Figure 2B). The molecular weight of this anticoagulant factor was estimated to be about 30 kDa under reduced conditions (Figure 3A). However, these results suggest that EC217 was partially purified at least by 95%. 

***Fibrinolytic assay***

The current study found that the fractions obtained by HPLC were assayed considering fibrinolytic activity of each fraction. Consequently, the EC217 of HPLC was demonstrated by increase of the clotting time after the addition either to the plasma citrate or fibrinogen solution. The results showed that the EC217 of HPLC were completely prevented fibrin clot formation indicating significant fibrinolytic activity (Table 2).

**Table1:** Results of PT assay on crud venom and venom fractions obtained from gel filtration based on 100 and 10 µg protein respectively

Step	Fraction (%)	Protein (mg)	Yield%(pr)	PT test (s)	INR	*P*-Value
Venom	100	230	92	29.9±5.90	2.31±0.31	*P*<0.01
EC1	32.82	65.7	34.34	12.34±0.50	1.12±0.04	NS
EC2	30.54	63.97	33.44	27.12±6.99	2.44±0.64	*P*<0.01
EC3	12.61	61.6	32.20	14.69±3.26	1.33±0.3	NS
Control				11±1.19	1	

NS: Indicates No significant. Control: The normal human plasma without venom sample in PT test

PT: Prothrombin Time

**Table2:** Results of PT and TT test on purified peptide (EC217)

Step	Protein (µg/ml)	PT assay(s)	INR for PT	TT assay(s)	*P*-Value
EC217	51	102±4.242	9.466±0.387	> 5min	*P* <0.001
Control	_	10.77±0.813	1	10.77±0.813	

Control: The PT assay of normal human plasma without addition of venom. Comparison for *P-* value was between control and EC217 peptide

PT: Prothrombin Time ; TT : Thrombin Time

## Discussion

Venoms from Viperidae family contain a large variety of proteins and peptides affecting the haemostatic system and a rich source of novel compounds that may have applications in medicine and biochemistry (21). This study has explained the central importance of a relatively simple procedure for the isolation of an anticoagulant factor from Iranian *E. carinatus *venom, denominated EC217 which was isolated and purified by a combination of gel filtration on Sephadex G-75 (Figure 1) and HPLC (Figure 2A). Previous studies showed that an anticoagulant was purified from *E. carinatus *venom in India by a three-step procedure comprised of gel filtration on Sephadex G-50, cation-exchange chromatography on Mono S, and C18, HPLC (22). Isolation of an anticoagulant factor from Iranian *Agkistrodon halys *venom, denominated AH22, which was isolated and purified by a combination of gel filtration on Sephadex G-50 and ion-exchange chromatography on DEAE-Sepharose (23). This study has investigated the initial Sephadex G-75 fractionation of the crude *E. carinatus *venom revealed three peaks (EC1-EC3) as shown in Figure 1 out of which fraction EC2 showed significant anticoagulation activity (Table1). The short form, EC was used as name of each fraction taking the first alphabet of snake species name *E. carinatus. *The numbers after EC indicating the fraction numbers. Further purification was carried out by RP-HPLC (Figure 2) which revealed 54 fractions out of which fraction EC217 showed anticoagulant and fibrinolytic activity. Finally, about 51 μg/ml of purified anticoagulant proteins (EC217) was obtained (Table 2). Snake venom toxins that prolong blood coagulation are proteins or glycoproteins that inhibit blood coagulation by different mechanisms. Some of these anticoagulant proteins such as Echicetin is a C-type lectin-like protein isolated from the venom of the saw-scaled viper (*E. carinatus*) acting as agonist/antagonist of platelet receptors (26-27). 

The measurement of plasma ACT and clot rate after activation with snake venoms and purified anticoagulant protein from crude *E. carinatus* venom was a fast and reproducible way to screen and classify venoms as procoagulant or anticoagulant (Table 1). We propose that fractions corresponding to EC2 contained anticoagulant proteins as determined by prothrombin time and fibrinolytic assays. Therefore, anticoagulant proteins from EC2 were isolated in these studies. EC2 was obtained using gel chromatography (Figure 1). One of the more significant findings to emerge from this study is that EC217 of HPLC corresponding to anticoagulant protein was pooled separately and purified using RP-HPLC (Figure 2A). In addition, EC217of HPLC acts as benign defibrinogenating agents to remove fibrinogen from the blood. The sole reason for the anticoagulation property of EC217 was thought to be based on its increase in the bleeding time can be influenced by the decrease in platelet count. Within minutes of administration of EC217, there was a depression in the fibrinogen concentration in plasma and within one hour the fibrinogen levels became very low and thus remained for more than 24 hr. These results suggest that the anticoagulant effect of EC217 of HPLC could be attributed to its fibrinolytic action on fibrinogen, forming fibrin monomers (non cross-linked fibrin), which could be rapidly removed from the circulation (27). *Purified peptide EC217* had significant anticoagulant activity on plasma or on fibrinogen. Previous studies clearly revealed snake venom toxins that prolong blood coagulation are proteins or glycoproteins with molecular masses ranging from 6 to 350 kDa (10, 12). Venoms from snake species belonging to the genus *Echis (E. multisquamatus, E. carinatus*) contain anticoagulant proteins. They are proteins with a molecular mass ranging from 5.2 to 30 kDa (10, 12). Some anticoagulant factors, along with their molecular weights, reported in the literature are: for example an acidic phospholipase A2 (EC-I-PLA2) has been purified from the Indian saw-scaled viper (*E. carinatus*) with the molecular wight of 16000 Da (15); while molecular weight of anticoagulant protein, Echicetin, from *E. carinatus* was reported to be 30 kDa (25)*.* In the present study, we also found that the molecular weight of the isolated peptide EC217 under denaturing conditions was about 30 kDa. Based on the present investigations, we concluded, this anticoagulant factor should belong to the low-molecular-weight group of these factors, on account of its molecular mass (30 kDa). Previous studies provided clear evidence that anticoagulant complex specifically inhibited the extrinsic tenase complex which named it as Echicetin. Echicetin itself does not activate washed platelets but inhibited platelet activation by vWF, thrombin, or alboaggregin A (25, 27). In this case the increase in the bleeding time can be influenced by the decrease in platelet count as well as by inhibition of vWf/thrombin platelet activation by Echicetin. C-type lectin-like protein was common in vipers venoms. The present findings seem to be consistent with other research which found that snake venoms with C-type lectin-like protein could have anticoagulant activity (15, 27). 

## Conclusion

*E. carinatus* venom was fractionated by means of gel filtration. Fraction II with anticoagulant activity was further purified by HPLC and finally purified peptide of EC217 was obtained. Fibrinogenolytic activity was assessed by PT test and fibrinolytic assay EC217 of HPLC was obtained after second chromatography of EC2 to show a single band at approximately 30 kDA regions. The following conclusions can be drawn from the present study that the venom of *E. carinatus* venom effectively inhibits the coagulation and fibrin clot formation and also their potential therapeutic use for occlusive arterial or venous thromboembolism.
